# A new species of *Proceratophrys* Miranda-Ribeiro, 1920 (Anura, Odontophrynidae) from Southern Amazonia, Brazil

**DOI:** 10.7717/peerj.12012

**Published:** 2021-09-10

**Authors:** Diego J. Santana, Leandro Alves da Silva, Anathielle Caroline Sant’Anna, Donald B. Shepard, Sarah Mângia

**Affiliations:** 1 Instituto de Biociências, Universidade Federal de Mato Grosso do Sul, Campo Grande, MS, Brazil; 2 Departamento de Sistemática e Ecologia, Universidade Federal da Paraíba, João Pessoa, PB, Brazil; 3 School of Biological Sciences, Louisiana Tech University, Ruston, LA, United States

**Keywords:** Horned-frogs, Systematics, *Proceratophrys korekore* sp. nov., DNA barcoding

## Abstract

Based on concordant differences in morphology, male advertisement call, and 16S mtDNA barcode distance, we describe a new species of *Proceratophrys* from southern Amazonia, in the states of Mato Grosso and Pará, Brazil. The new species is most similar to *P. concavitympanum* and *P. ararype* but differs from these species by its proportionally larger eyes and features of the advertisement call. Additionally, genetic distance between the new species and its congeners is 3.0–10.4% based on a fragment of the 16S rRNA gene, which is greater than the threshold typically characterizing distinct species of anurans. Using an integrative approach (molecular, bioacoustics, and adult morphology), we were able to distinguish the new species from other congeneric species. The new species is known only from the type locality where it is threatened by illegal logging and gold mining as well as hydroelectric dams.

## Introduction

Integrative taxonomic approaches are considered paramount to describe new species (Padial et al., 2010). This is especially true for taxa in megadiverse regions, where new species are usually found (Toledo & Batista, 2012; Moura et al., 2018), and within speciose genera, where species are often misidentified (Fouquet et al., 2007). Such a diversity scenario occurs in Amazonia, the largest tropical forest in the world, where many anuran species are still unnamed (Funk, Caminer & Ron, 2011; Rojas et al., 2018). Many Amazonian species are considered widespread, but studies using integrative approaches have unveiled new species and lineages covering much smaller areas than previously known (Fouquet et al., 2007; Funk, Caminer & Ron, 2011; Ferrão et al., 2016). Focusing taxonomic efforts to reveal this hidden diversity is important for properly documenting Earth’s biodiversity (Brito, 2010) and for illuminating diversification patterns. Undescribed species occurring in megadiverse regions may also face threats that lead them to become extinct before they can be formally described (Lees & Pimm, 2015), especially in regions where anthropogenic disturbance has drastically changed the landscape, such as with hydroelectric dams in Amazonia (Silva et al., 2018).

The Amazon forest harbors several populations of *Proceratophrys* Miranda-Ribeiro, 1920, which are historically identified as *P. concavitympanum* Giaretta, Bernarde & Kokubum, 2000 or *P. rondonae* Prado & Pombal, 2008 (Prado & Pombal, 2008; Bernardo, Matiazzi & Guerra-Fuentes, 2012). These species are easily distinguished because *P. rondonae* has well-developed palpebral appendages, which are not present in *P. concavitympanum* (Prado & Pombal, 2008). Therefore, all populations of *Proceratophrys* across Amazonia without developed eyelid appendages have been called *P. concavitympanum*, yielding a widespread distribution for this species (Bernardo, Matiazzi & Guerra-Fuentes, 2012). Nevertheless, only two populations identified as *P. concavitympanum* have molecular data available on GenBank: one from a Cerrado region (Palmas municipality, Tocantins state; Amaro, Pavan & Rodrigues, 2009) and the other from Amazonia (Aripuanã municipality, Mato Grosso state; Mângia et al., 2018). The population from Palmas is more closely related to *P. ararype* from relictual forests within Caatinga in northeastern Brazil (Mângia et al., 2018). Once the authors found that individuals from Palmas are not *P. concavitympanum*, they suggested that other populations of *Proceratophrys* throughout Amazonia are in need of further revision.

During fieldwork along the Teles Pires River in southern Amazonia, we collected specimens of *Proceratophrys* previously identified as *P. concavitympanum* (Ávila, Kawashita-Ribeiro & Morais, 2011). Here, we combine morphological, bioacoustical, and mtDNA evidence to elucidate the taxonomic status of this population and describe it as a new species.

## Materials & methods

### Fieldwork

We collected seven adult specimens (five males and two females) and five juveniles during visual searches in two sites along the Teles Pires River, Brazil: Paranaíta municipality, Mato Grosso state, on the west bank (09°18′57.96″ S, 56°47′33.53″ W, 250 m a.s.l.; *datum* = SAD69), and Jacareacanga municipality, Pará state, on the east bank (09°19′1.00″ S, 56°46′35.76″ W, 200 m a.s.l.; *datum* = SAD69). We euthanized individuals in a liquid solution of 2% lidocaine chlorhydrate, following Conselho Federal de Biologia resolution CFBio n°148/2012 (CFBio, 2012), fixed them in 10% formalin, and preserved them in 70% alcohol. Prior to fixation, we collected tissue samples (muscle and liver) and stored them in individual tubes of 100% ethanol. Voucher specimens and tissues are deposited in the Coleção Zoológica da Universidade Federal de Mato Grosso do Sul (ZUFMS-AMP), Campo Grande municipality, Mato Grosso do Sul state, Brazil. Collection permits for this study were issued by ICMBIO (SISBio 45889-1).

### Morphology

Specimens used in the description and examined for comparison are housed in 11 herpetological collections in Brazil (see Appendix 1). We followed the terminology for morphological characters of Prado & Pombal (2008) and Brandão et al. (2013). We followed Prado & Pombal (2008) for the 13 morphometric variables: snout–vent length (SVL), head length (HL), head width (HW), distance from the interocular crest to the tip of snout (DICS), internarial distance (IND), eye–nostril distance (END), eye diameter (ED), upper eyelid width (UEW), interorbital distance (IOD), thigh length (THL), tibia length (TL), foot plus tarsus length (FL), and forearm and hand length (FHL). All measurements were made by DJS using a digital caliper (0.01 mm precision, which we rounded to one decimal in order to avoid imprecision). We determined the sex of each individual by the presence of vocal slits in males and their absence in females. Finally, for the eyelid pattern tubercles formulae, we followed Brandão et al. (2013), which takes into account the number of anterior and posterior tubercles in each eyelid (right and left), the extension of the medial tubercle, expressed by the side (L or R), the number of anterior tubercles, the extension of the elongated medial tubercle (expressed in proportion), and the number of posterior tubercles.

### Bioacoustics

We recorded the advertisement call of the paratype ZUFMS-AMP13680 at Jacareacanga (the type locality), and analyzed a total of eight advertisement calls. We used a Tascam DR-44 digital recorder to record the calls around 20:00 h (air temperature 24.3 °C; humidity 86%) recorded at 44.1 kHz with 16-bit resolution in *.wav* format. We analyzed calls in Raven Pro v1.5 for Mac (Bioacoustics Research Program, 2014) and constructed audio spectrograms in R using the package *‘seewave’* (Sueur, Aubin & Simonis, 2008) with the following parameters: FFT window width = 256, frame = 100, overlap = 75, and flat top filter. We analyzed the acoustic parameters: call duration, pulse number per call, pulse rate, which was measured as the ratio of the absolute number of pulses and the absolute duration in which these pulses were emitted, and dominant frequency. Terminology of call descriptions follows Köhler et al. (2017), and values are reported as average ± SD (minimum–maximum). We deposited the sound recording in the acoustic collection of the Fonoteca Mapinguari da Universidade Federal de Mato Grosso do Sul (MAP-V 329).

### Phylogenetic inference and genetic distances

We sequenced fragments of the 16S ribosomal RNA mitochondrial gene from three individuals of the new species and four individuals of *P. strussmannae* from its type locality (Document S1). We extracted genomic DNA from liver samples using the phenol-chloroform protocol of Sambrook & Russell (2001). We used the 16Sa/16Sb primer pair of Palumbi et al. (1991), following PCR conditions described by Costa et al. (2016). PCR reactions consisted of 1× buffer, dNTPs at 0.2 mM, each primer at 0.2 μM, MgCl_2_ at two mM, one U Taq polymerase, and two μl of template DNA, in a total reaction volume of 25 μl. We used the following PCR cycling program: 94 °C for 2 min, followed by 35 cycles of 94 °C for 30 s, 59 °C for 1 min, and 72 °C for 1 min, and a final 5 min extension at 72 °C. We purified PCR products with Ethanol/Sodium Acetate and sequenced them on an ABI 3730XL DNA Analyzer (Applied Biosystems, Foster City, CA, USA). Resulting sequences were edited and aligned using Geneious v9.1.2 with the MUSCLE algorithm using default parameters (Edgar, 2004). We aligned our 16S sequences with 16S sequences of other species of *Proceratophrys* and with the outgroups *Odontophrynus* spp., *Macrogenioglottus alipioi, Cycloramphus acangatan* and *Thoropa miliaris*, which are available in GenBank (Document S1). The final aligned dataset used in all analyses comprised 421 base pairs (bp) of 16S. We used the Bayesian Information Criterion in jModelTest (Darriba et al., 2012) to determine that HKY+I+G was the best model of nucleotide substitution for our 16S data set.

We performed a Bayesian phylogenetic analysis of 16S using BEAST v.2.6.3 (Bouckaert et al., 2019) for 50 million generations, sampling every 5,000 steps using a Yule Process tree prior. We checked for stationarity by visually inspecting trace plots and ensuring that all values for effective sample size were above 200 in Tracer v1.7.1 (Rambaut et al., 2018). The first 10% of sampled genealogies were discarded as burn-in, and the maximum clade credibility tree with median node ages was calculated with TreeAnnotator v2.6.3 (Bouckaert et al., 2019). We also calculated sequence divergence (uncorrected p-distance) among species/individuals using MEGA v10.1.1 (Kumar et al., 2018). In order to explore the relationship among haplotypes, we estimated haplotype networks among species closely related to the new species (*P. concavitympanum* clade) for the 16S mtDNA gene in POPART (Leigh & Bryant, 2015) using the median-joining network method. We identified each species using different colors in the haplotype network.

### Nomenclatural acts

The electronic edition of this article conforms to the requirements of the amended International Code of Zoological Nomenclature, and hence the new names contained herein are available under that Code of this article. This published work and the nomenclatural acts it contains have been registered in ZooBank, the online registration system for the ICZN. The LSID (Life Science Identifier) for this publication is: LSIDurn:lsid:zoobank.org:pub:4077F2CC-A0B1-49AC-B562-2FE7CC929668. The electronic edition of this work was published in a journal with an ISSN, has been archived, and is available from the following digital repository: www.peerj.com/.

## Results

**Figure 1:**
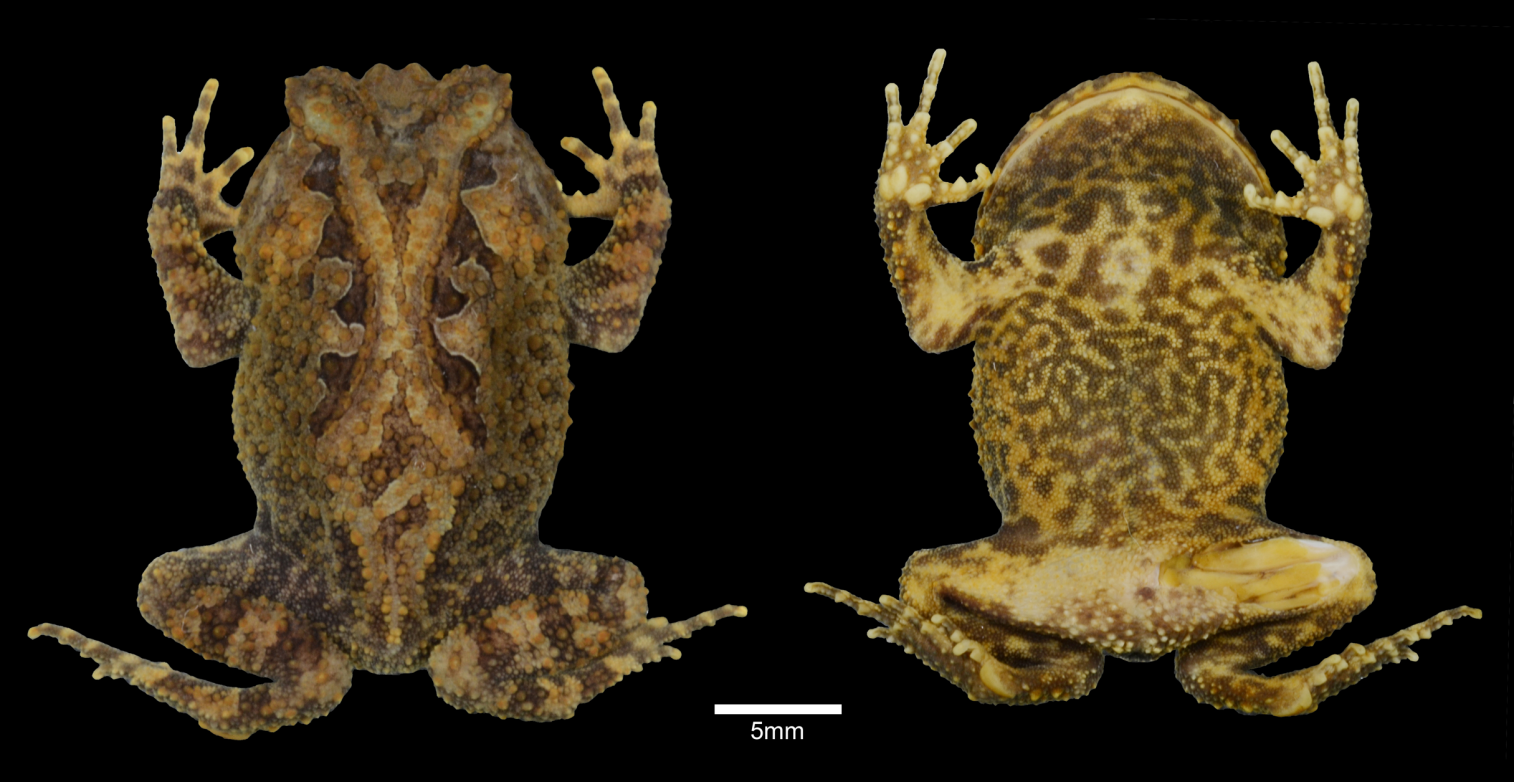
Holotype of *Proceratophrys korekore* sp. nov. (ZUFMS-AMP08100). (Left) Dorsal view of the body; and (right) ventral view of the body.

**Figure 2:**
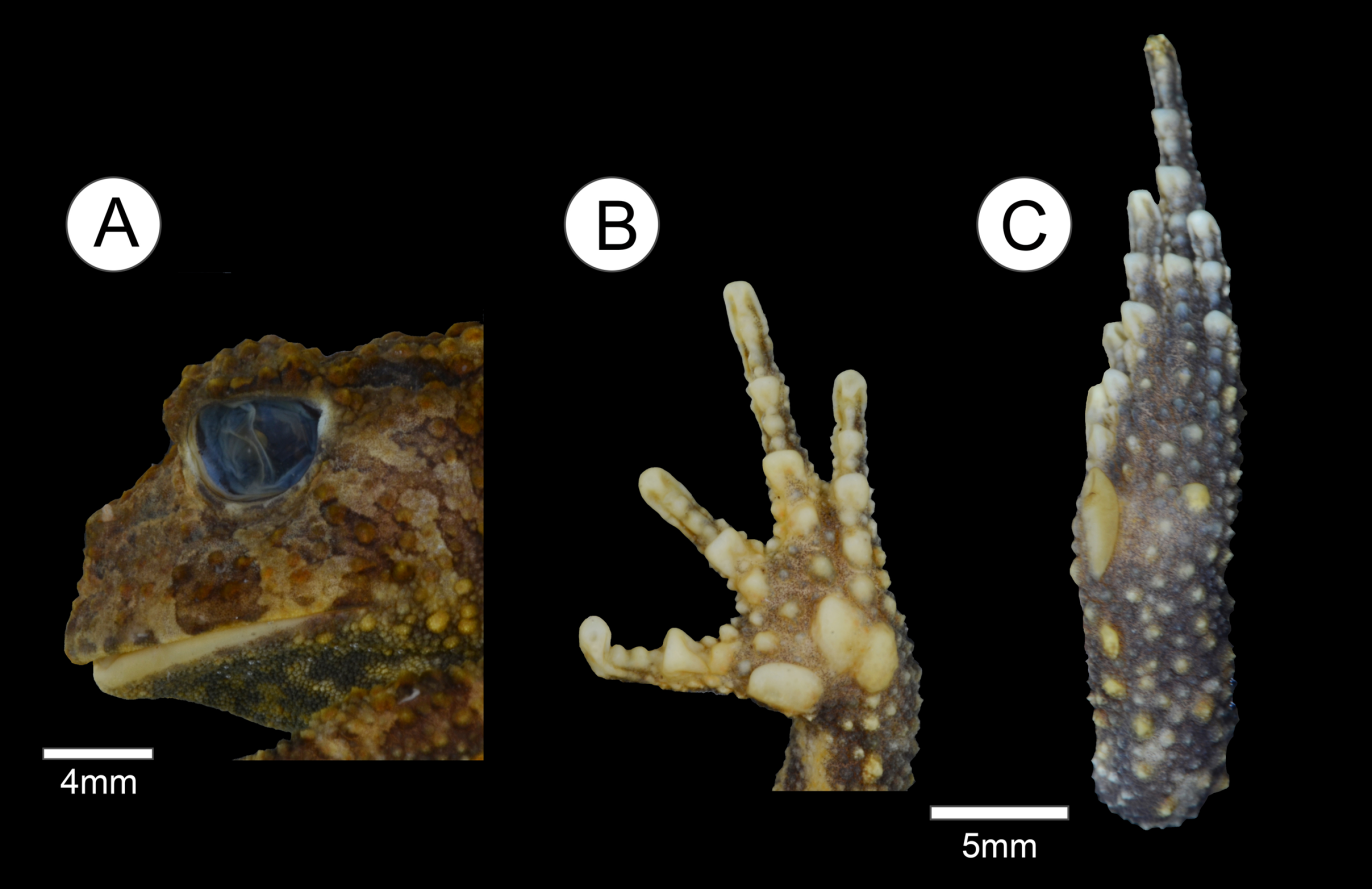
Holotype of *Proceratophrys korekore* sp. nov. (ZUFMS-AMP08100). (A) Head lateral view; (B) ventral view of right hand; and (C) ventral view of right foot.

**Figure 3:**
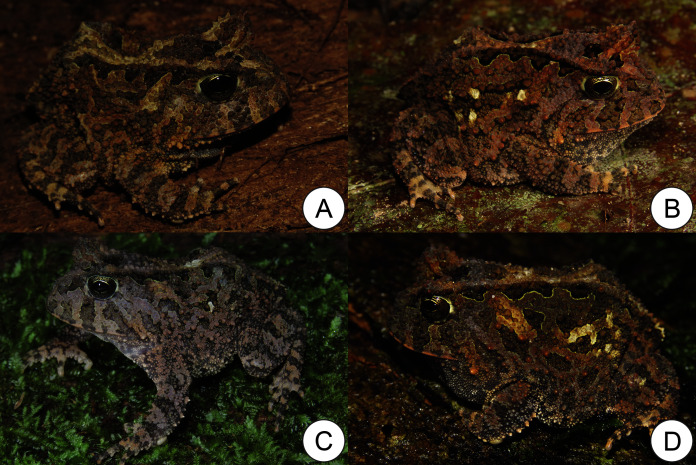
Live specimens of *Proceratophrys korekore* sp. nov. from Jacareacanga (type locality), Pará state, Brazil. (A) Paratype adult male (ZUFMS-AMP08105), (B) paratype adult male (ZUFMS-AMP13681), (C) unvouchered adult female, and (D) unvouchered adult male.

**Figure 4:**
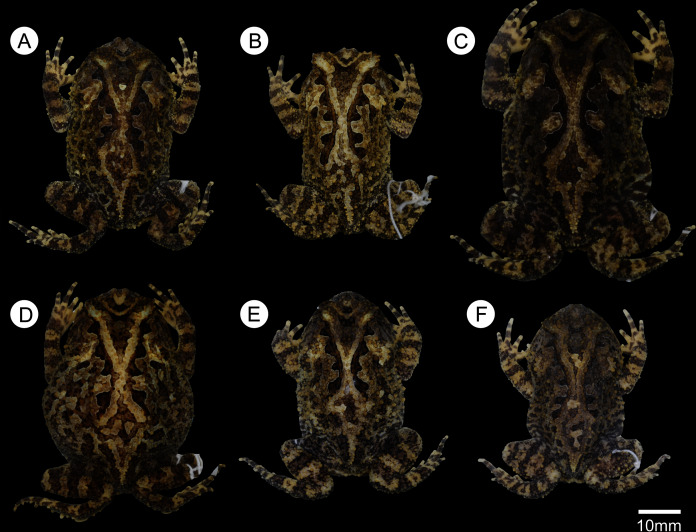
Dorsal color variation in preservative among specimens from type series. (A) ZUFMS-AMP 08102, (B) ZUFMS-AMP 08105, (C) ZUFMS-AMP 08116, (D) ZUFMS-AMP 08117, (E) ZUFMS-AMP 13680, (F) ZUFMS-AMP 13681.

***Proceratophryskorekore* sp. nov.** (Fig. 1–4)

urn:lsid:zoobank.org:pub:4077F2CC-A0B1-49AC-B562-2FE7CC929668

*Proceratophrys concavitympanum*Ávila, Kawashita-Ribeiro & Morais (2011) (part)

**Holotype.** ZUFMS-AMP08100, adult male, from Jacareacanga municipality, Pará state, Brazil (09°19′1.00″ S, 56°46′35.76″ W, 200 m a.s.l.; *datum* = SAD69), collected on 08 November 2016, by L.A. Silva.

**Paratypes.** ZUFMS-AMP08098, juvenile from the type locality, collected on 30 May 2017, by L.A. Silva and E.A. Pereira; ZUFMS-AMP08099, juvenile from the type locality, collected on 25 May 2017, by L.A. Silva and E.A. Pereira; ZUFMS-AMP08101, ZUFMS-AMP08103, two juveniles, and ZUFMS-AMP08102, adult male, collected along with the holotype by L.A. Silva; ZUFMS-AMP08104, juvenile, from the type locality, collected on 22 May 2016, by L.A. Silva; ZUFMS-AMP08105, adult male, from the type locality, collected 26 February 2017, by L.A. Silva; ZUFMS-AMP08116, adult female, from Paranaíta municipality, Mato Grosso state, Brazil (09°39′47″ S, 56°28′36″ W, 200 m a.s.l.; *datum* = SAD69), collected on 08 November 2016, by L.A. Silva; ZUFMS-AMP08117, adult female, from Paranaíta municipality, Mato Grosso state, Brazil (09°39′47″ S, 56°28′36″ W, 200 m a.s.l.; *datum* = SAD69), collected on 24 February 2016, by M.O. Neves; ZUFMS-AMP13680, adult male, from the type locality, collected on 2 May 2018, by L.A. Silva and H. Folly; ZUFMS-AMP13681, adult male, from the type locality, collected on 14 May 2018, by L.A. Silva and H. Folly. CFBH 20675, 20676, 20678, adult males, and CFBH 20677, adult female, from Paranaíta municipality, Mato Grosso state, Brazil (9°39′52.99″ S, 56°28′36.12″ W, 270 m a.s.l.; *datum* = WGS84), collected on 24 November 2008, collector not informed; ZUEC 14874, 14875, 14950 adult females, and ZUEC 14876, adult male, from Paranaíta municipality, Mato Grosso state, Brazil (9°39′52.99″ S, 56°28′36.12″ W, 270 m a.s.l.; *datum* = WGS84), collected on 21 February 2009, by L.F. Toledo, N.R. Silva, O. Araújo and I. Prates; ZUEC 16011–16014, 16719, juveniles, and ZUEC 16015, adult female, from Paranaíta municipality, Mato Grosso state, Brazil (9°39′52.99″ S, 56°28′36.12″ W, 270 m a.s.l.; *datum* = WGS-84), collected on 11 June 2009, by L.F. Toledo, O. Araujo, R.S. Nelson and P.L.M. Fontana; UFMT-A 7534, juvenile from UHE Foz do Apiacás, Paranaíta municipality, Mato Grosso state, Brazil (9°22′58.44″ S, 57°3′47.16″ W, 270 m a.s.l.; *datum* = WGS84), collected on 01 June 2008, collector not informed; UFMT-A 7963, juvenile, from UHE Foz do Apiacás, Paranaíta municipality, Mato Grosso state, Brazil (9°22′58.44″ S, 57°3′47.16″ W, 270 m a.s.l.; *datum* = WGS-84), collected on 16 September 2008, collector not informed; UFMT-A 9882, 10038, juveniles, from UHE Foz do Apiacás, Paranaíta municipality, Mato Grosso state, Brazil (9°22′58.44″ S, 57°3′47.16″ W, 270 m a.s.l.; *datum* = WGS84), collected on 20 April 2009, collector not informed; UFMT-A 9990, adult female, from UHE Foz do Apiacás, Paranaíta municipality, Mato Grosso state, Brazil (9°22′58.44″ S, 57°3′47.16″ W, 270 m a.s.l.; *datum* = WGS84), collected on 20 September 2009, collector not informed; UFMT-A 10041, 10046, 10054, juveniles, from UHE Foz do Apiacás, Paranaíta municipality, Mato Grosso state, Brazil (9°22′58.44″ S, 57°3′47.16″ W, 270 m a.s.l.; *datum* = WGS84), collected on 17 April 2009, collector not informed; UFMT-A 10067, juvenile, from UHE Foz do Apiacás, Paranaíta municipality, Mato Grosso state, Brazil (9°22′58.44″ S, 57°3′47.16″ W, 270 m a.s.l.; *datum* = WGS84), collected on 19 April 2009, collector not informed; UFMT-A 10109, juvenile, from UHE Foz do Apiacás, Paranaíta municipality, Mato Grosso state, Brazil (9°22′58.44″ S, 57°3′47.16″ W, 270 m a.s.l.; *datum* = WGS84), collected on 22 April 2009, collector not informed.

**Diagnosis.** The new species can be distinguished by the following combination of traits: (1) medium size (39.8–44.1 mm SVL in adult males; 43.8–57.6 mm SVL in adult females); (2) upper eyelid border with fused and small pointed warts; (3) proportional measurements ED/END 1.1–1.3; (4) presence of a single row of tubercles of different sizes bordered with some sparse tubercles on the forearm; (5) call duration of 0.162–0.332 s; 18–31 pulses/call; pulse rate of 96.4–111.1 pulses/s.

**Comparison with other species.***Proceratophrys korekore* sp. nov. differs from *P. appendiculata*, *P. belzebul*, *P. boiei*, *P. gladius*, *P. itamari*, *P. izecksohni*, *P. laticeps*, *P. mantiqueira*, *P. melanopogon*, *P. moehringi*, *P. paviotii*, *P. phyllostomus*, *P. pombali*, *P. renalis*, *P. rondonae*, *P. sanctaritae*, *P. subguttata*, and *P. tupinamba* by the absence of a single unicuspidate palpebral appendage (a single and long unicuspidate palpebral appendage in all species, except in *P. rondonae*, which has a single and short multi-cuspidate palpebral appendage). In addition, *P. korekore* sp. nov. can be distinguished from *P. appendiculata*, *P. belzebul*, *P. gladius*, *P. itamari*, *P. izecksohni*, *P. laticeps*, *P. mantiqueira*, *P. melanopogon*, *P. moehringi*, *P. phyllostomus*, *P. pombali*, *P. sanctaritae*, *P. subguttata*, and *P. tupinamba* by lacking a rostral appendage (present in those species). *Proceratophrys korekore* sp. nov. differs from *P. avelinoi*, *P. bigibbosa*, *P. brauni*, and *P. palustris* by lacking postocular swellings (present in these species). *Proceratophrys korekore* sp. nov. has fused small pointed warts on the upper eyelid border (short, fused, and not pointed in *P. ararype*; small, rounded, and not fused in *P. cururu* and *P. rotundipalpebra*; slightly fused without appendage in *P. branti*, *P. huntingtoni*, *P. vielliardi*, and *P. moratoi*; conical and pointed in *P. bagnoi*; enlarged, pointed, and with the largest tubercle in the middle more projected than lateral tubercles in *P. minuta*; small and rounded in *P. redacta*; multiple short and pointed expansions in *P. schirchi*). From *P. bagnoi*, *P. concavitympanum*, *P. dibernardoi*, and *P. goyana*, *P. korekore* sp. nov. differs by the presence of a single row of tubercles of different sizes bordered with some sparse tubercles on the forearm (two well-delimited rows of tubercles in *P. bagnoi*, *P. concavitympanum*, and *P. dibernardoi*; tubercles not organized in rows in *P. goyana*). From *P. concavitympanum*, *P. korekore* differs by its proportionally larger eyes ED/END 1.1–1.3 (ED/END 0.8–1.0 in *P. concavitympanum*). From *P. salvatori*, *P. korekore* sp. nov. differs by the presence of an ocular-dorsal ridge of warts (lack of ocular-dorsal ridge of warts in *P. salvatori*).

*Proceratophrys korekore* sp. nov. also differs from *P. ararype* by the shorter duration and lower number of pulses/call in the advertisement call (0.162–0.332 s, 18–31 pulses/call *versus* 0.374–0.648 s, 38–65 pulses/call, respectively). The lower pulse rate differentiates *P. korekore* sp. nov. (96.4–111.1 pulses/s) from *P. moratoi* (69–86 pulses/s), and the lower dominant frequency (861.3 Hz) from *P. ararype* and *P. moratoi* (1,033.6–1,378.1 Hz and 1,153–1,594 Hz, respectively).

**Description of the holotype.** Head wider than long, head length 70% of SVL, snout semi-circular in dorsal and ventral views, obtuse and slightly vertical in profile; nares elliptical and prominent, canthal crests marked, prominent, and covered by small tubercles; no preocular crests; eyes directed anterolaterally, eye diameter 38% of head length and 95% of upper eyelid width; eyelid with short warts, with the contact point between the ocular-dorsal ridge of warts and the external eyelid margin tubercles in the posterior third region, eight warts on the left eyelid and six on the right (L 2, 2/5, 5; R 2, 2/5, 3), with one more prominent; presence of one row of tubercles on the eyelid; indistinct tympanum; vomerine teeth in two short rows between and above the choanae; frontoparietal crests well developed; region between frontoparietal crests shallow; interocular ridge of warts organized in a row, markedly curved; ocular-dorsal ridge of warts complete from the eyes to the sacral diapophysis, and discontinued to the coccyx region. Dorsal surface, including flanks, arms and legs, with various warts of different sizes and shapes, a single row of tubercles in different sizes bordered with some sparse tubercles on the forearm; ventral surfaces, except hands and feet and cloacal region, covered by numerous small, rounded, uniform warts. Finger lengths IV > II > I > III (Fig. 2B); interdigital webbing absent; inner metacarpal tubercle large and elliptical; outer metacarpal divided in two parts, both internal and external are elliptical; scarce small, rounded supernumerary tubercles; subarticular tubercles large, rounded, but grooved anteriorly and posteriorly. Thigh length longer than tibia length (THL/TL = 1.1), the sum of thigh and tibia lengths 81% of SVL; toe lengths I > II > V > III > IV; inner metatarsal tubercle long, elliptical, spatulated; outer metatarsal tubercle small, rounded; scarce small, rounded supernumerary tubercles; subarticular tubercles large, nearly rounded, grooved anteriorly and posteriorly.

**Measurements of the holotype (mm).** SVL 43.4; HL 15.7; HW 22.0; DICS 10.8; IND 3.9; END 4.6; ED 6.0; UEW 6.3; IOD 3.2; THL 18.3; TL 16.7; FL 25.4; FHL 23.2 (Table 1).

**Table 1:** Morphometric measurements (mm) for the type series of *Proceratophrys korekore* sp. nov.

Measurement	ZUFMS-AMP 8100 (Holotype)	Males (*n* = 12)			Females (*n* = 9)		
		Mean	SD	Range	Mean	SD	Range
SVL	43.35	43.7	3.6	39.8–53.5	48.7	9.3	33.0–57.6
HL	15.67	15.3	1.3	13.7–18.9	17.4	3.3	12.5–21.0
HW	21.97	20.6	1.4	18.1–22.7	23.0	4.4	16.6–28.1
DICS	10.74	10.1	0.9	8.6–11.0	11.3	1.8	8.9–14.0
IND	3.86	2.9	0.5	2.2–3.9	3.1	0.8	2.2–4.2
END	4.55	4.1	0.4	3.5–4.7	4.3	0.6	3.5–5.4
ED	5.96	4.8	0.6	4.1–6.0	4.9	0.7	4.2–6.2
UEW	6.27	6.2	0.6	5.4–7.3	6.1	1.0	4.9–7.7
IOD	3.15	6.0	2.5	3.0–10.0	7.3	2.0	3.9–9.8
THL	18.34	17.9	1.2	15.5–20.0	20.1	3.8	14.6–25.0
TL	16.71	16.9	0.9	15.2–18.3	18.3	3.3	13.7–22.1
FL	25.36	24.7	1.5	22.0–26.9	26.8	5.0	19.1–32.5
FH	23.23	22.2	1.4	19.9–24.3	24.0	4.6	16.7–30.5

**Note:**

Measurements acronyms: SVL, snout–vent length; HL, head length; HW, head width; DICS, distance from the interocular crest to the tip of snout; IND, internarial distance; END, eye–nostril distance; ED, eye diameter; UEW, upper eyelid width; IOD, interorbital distance; THL, thigh length; TL, tibia length; FL, foot plus tarsus length; FHL, forearm and hand length.

**Color of the holotype in preservative.** Dorsal background color brown. Area delimited by the ocular-dorsal ridge of warts light brown, bordered by four dark brown triangular blotches on each side. Two light brown bands from the eye to the upper lip. From two to three transverse dark-brown bars on the fingers and toes. Ventral surface cream with mottling dark brown, becoming darkish in the gular region (Fig. 1B).

**Variation.** Color variation is related to the size and shape of blotches, and background color varies from light to dark brown in life (Fig. 3) and in preservative (Fig. 4). Some individuals do not have a complete ocular-dorsal ridge of warts, and it varies within individuals with some more complete than others (Fig. 4). Within the collected type series, females are larger than males. Measurements of the type series are provided in Table 1.

**Figure 5:**
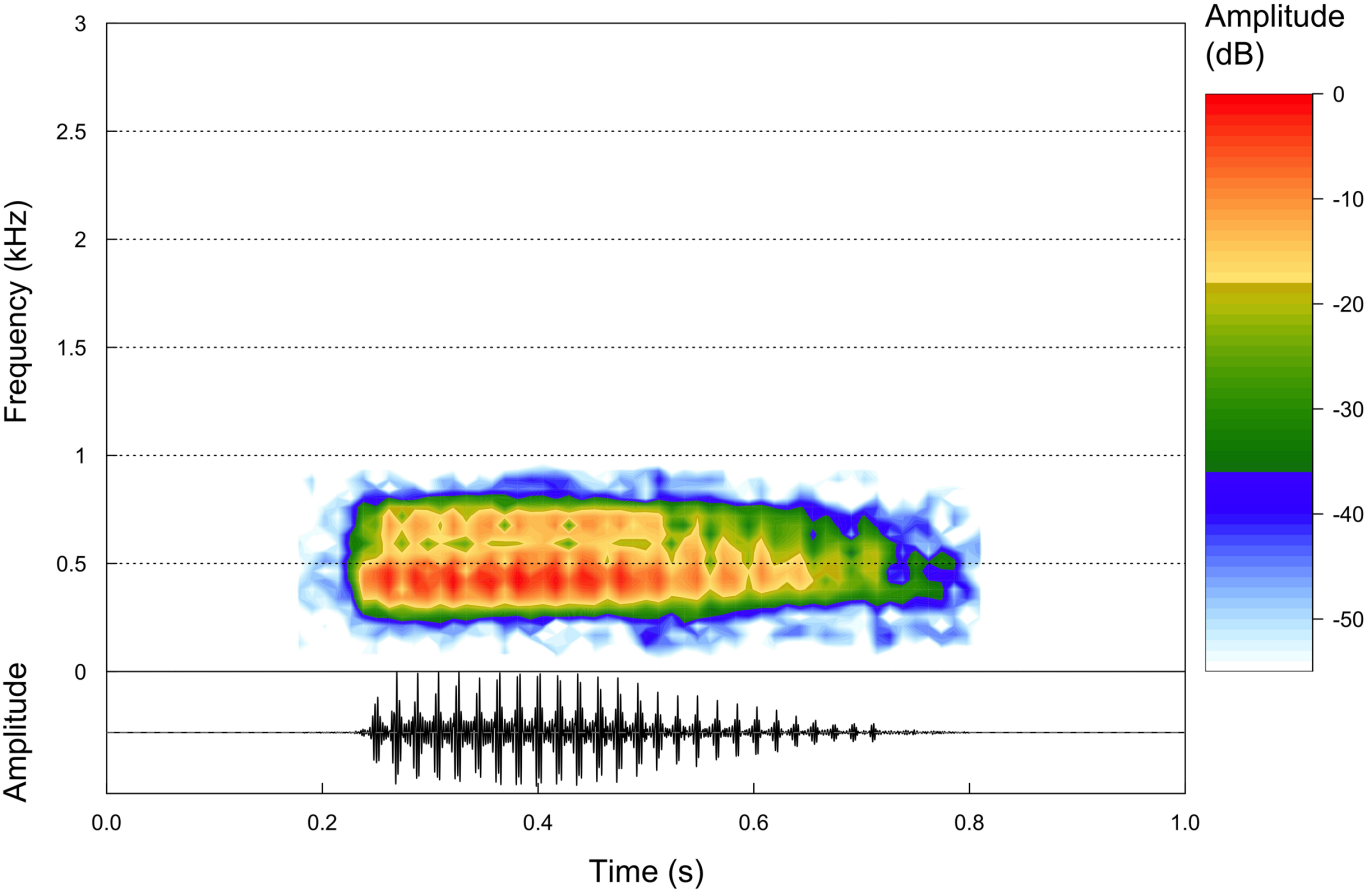
Advertisement calls. Oscilogram and Spectogram of an advertisement call of the paratype of *Proceratophrys korekore* sp. nov. (ZUFMS-AMP13680) from the type locality (air temperature 24.3 °C; SVL 42.94 mm).

**Advertisement call.** Based on eight calls from one paratype male (ZUFMS-AMP13680) recorded at the type locality, the advertisement call of *P. korekore* sp. nov. consists of a single pulsed note (Fig. 5) with duration of 0.162–0.332 s (0.251 ± 0.053 s), emitted sporadically with 18–32 pulses/call (25.0 ± 4.4), at a rate of 96.4–111.1 pulses/s (103.9 ± 5.9 pulses/s), and at a dominant frequency of 861.3 Hz, without variation (Table 2).

**Table 2:** Acoustic parameters of the advertisement calls of species from the *Proceratophrys concavitympanum* clade.

Species	Duration (s)	Pulses/call	Pulses/s	Dominant frequency (Hz)	Location	Source
*P. korekore* sp. nov. 1 male, 8 calls	0.251 ± 0.053 (0.162–0.332)	25.0 ± 4.4 (18–32)	103.9 ± 5.9 (96.4–111.1)	861.3	Jacareacanga, MT	This study
*P. ararype* 3 males, 120 calls	0.498 ± 0.06 (0.374–0.648)	49.4 ± 6.6 (38–65)	99 ± 1.5 (95.7–102.7)	1,167.2 ± 76.8 (1,033.6–1,378.1)	Crato, CE	Mângia et al. (2018)
*P. concavitympanum* 3 males, 33 calls	0.367 ± 0.06 (0.230–0.500)	38.7 ± 7.4 (23–51)	106.3 ± 3.1 (100–112.3)	948.2 ± 66.7 (851.0–1,116.4)	Aripuanã, MT	Santana et al. (2010)
*P. concavitympanum* 1 male, 13 calls	0.278 ± 0.04 (0.178–0.326)	30.9 ± 4.8 (19–37)	110.9 ± 5.2 (100.7–119.3)	819.2 ± 62.2 (754.3–874.5)	Espigão do Oeste, RO	Santana et al. (2010)
*P. moratoi* 4 males, 126 calls	0.245 ± 0.03 (0.185–0.307)	20.5 ± 2.5 (15–26)	81–85	1,343.0 ± 73.7 (1,174–1,444)	Itirapina, SP	Brasileiro, Martins & Jim (2008)
*P. moratoi* 2 males, 59 calls	0.207 ± 0.02 (0.146–0.238)	17.5 ± 1.5 (12–20)	82–84	1,348.7–86.6 (1,153–1420)	Botucatu, SP	Brasileiro, Martins & Jim (2008)
*P. moratoi* 2 males, 44 calls	0.232 ± 0.02 (0.181–0.268)	19 ± 3.0 (14–23)	77–86	1,440 ± 50 (1,406–1,594)	Ituiutaba, MG	Martins & Giaretta (2012)
*P. moratoi* 7 males, 148 calls	0.253 ± 0.04 (0.179–0.335)	19 ± 2.0 (14–23)	69–78	1,327 ± 108 (1,219–1,464)	Uberlândia, MG	Martins & Giaretta (2012)

**Note:**

Values are presented as mean ± SD (range).

Among the described advertisement calls of the closely related species (*P. ararype*, *P. concavitympanum*, *P. korekore* sp. nov., *P. moratoi*, *P. salvatori* and *P. strussmannae*), *P. korekore* sp. nov. has a shorter call duration than *P. ararype* (0.374–0.648 s) (Mângia et al., 2018). The pulse rate of *P. korekore* sp. nov. is greater than *P. moratoi* (69–103 pulses/s) and *P. salvatori* (54–61 pulses/s) (Magalhães et al., 2020). Although the dominant frequency did not vary in *P. korekore* sp. nov., probably due to few calls and a single individual recorded, this parameter is lower than *P. ararype* (1,034–1,378 Hz), *P. moratoi* (1,153–1,594 Hz) and *P. salvatori* (1,572–1,875 Hz) (Mângia et al., 2018; Magalhães et al., 2020). The call parameters of *P. korekore* sp. nov. are similar to *P. concavitympanum* (Santana et al., 2010), and we were unable to compare with *P. strussmannae* because its advertisement call is not described.

**Figure 6:**
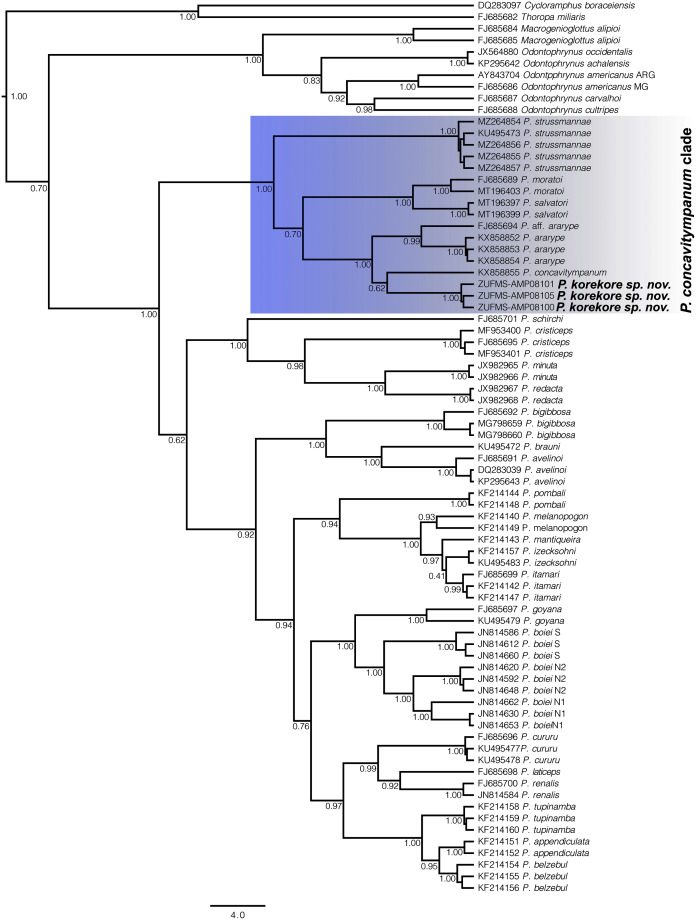
Gene tree chronogram. Phylogenetic analysis of the 16S mtDNA gene for the 26 species of the genus *Proceratophrys*. Nodes are labeled with the Bayesian posterior probability. Scale bar in million years.

**Figure 7:**
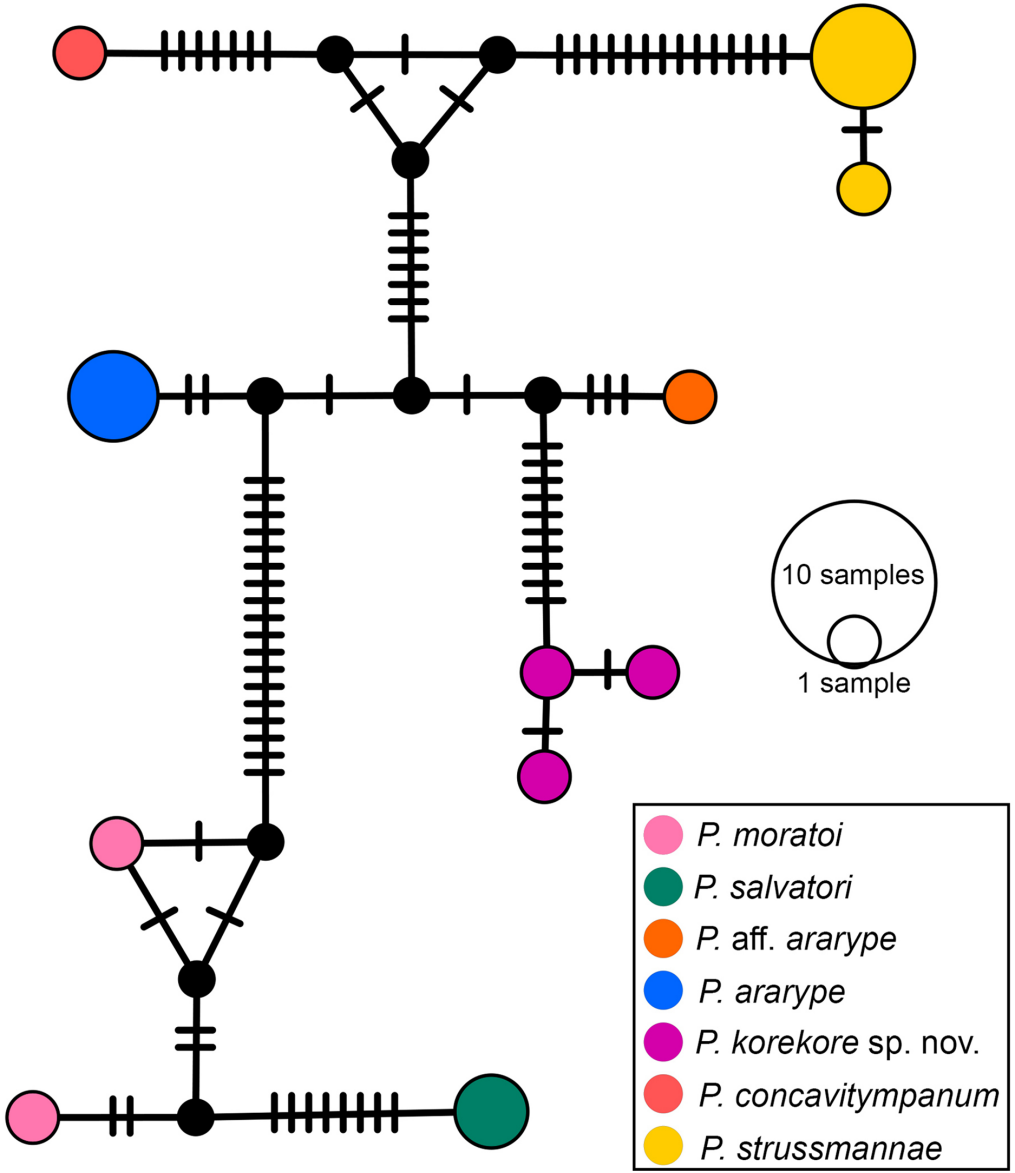
Haplotype network. Median-joining haplotype network of specimens from *P. concavitympanum* clade based on 16S mtDNA. Each haplotype is represented by a circle whose area is proportional to its frequency. Traits indicate additional mutational steps for branches with more than one mutation. Different colors indicate species-level units. The black dots are median vectors (hypothesized sequences).

**Phylogenetic analysis.** Our 16S tree confidently placed *P. korekore* sp. nov. as the sister taxon of *P. concavitympanum*, within a well-supported clade including *P. strussmannae*, *P. salvatori*, *P. moratoi*, and *P. ararype* (Fig. 6), hereafter called the *P. concavitympanum* clade. For some deeper nodes within *Proceratophrys*, our tree had low posterior probabilities, but this is expected given it is based on a single mtDNA locus utilized for barcoding species. Average sequence divergence between the new species and congeners ranges from 3.0% (*P*. aff. *ararype*) to 10.4% (*P. redacta*) (Table 3; Document S2). Furthermore, the mitochondrial haplotype network (Fig. 7) shows seven distinct mitochondrial lineages and no haplotype sharing between species of the *P. concavitympanum* clade.

**Table 3:** Mean p-distance of *Proceratophrys korekore* sp. nov. to other species of the genus.

Species	*p*-distance	Species	*p*-distance
*P. ararype*	3.5	*P. izecksohni*	7.1
*P*. aff. *ararype*	3.0	*P. laticeps*	7.1
*P. appendiculata*	6.8	*P. mantiqueira*	7.2
*P. avelinoi*	7.0	*P. melanopogon*	6.9
*P. belzebul*	7.3	*P*. aff. *melanopogon*	6.8
*P. bigibbosa*	7.1	*P. minuta*	10.0
*P. boiei* N1	7.0	*P. moratoi*	6.9
*P. boiei* N2	8.3	*P. pombali*	7.2
*P. boiei* S	7.1	*P. redacta*	10.4
*P. brauni*	7.4	*P. renalis*	9.0
*P. concavitympanum*	4.2	*P. salvatori*	6.5
*P. criticeps*	9.8	*P*. cf. *salvatori*	6.5
*P. cururu*	7.9	*P. schirchi*	9.1
*P. goyana*	7.2	*P. strussmannae*	6.3
*P. itamari*	7.0	*P. tupinamba*	6.7

**Figure 8:**
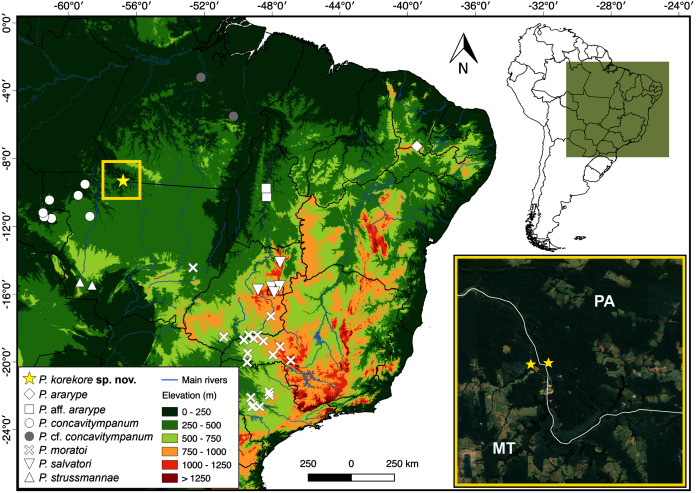
Geographic distribution of species from *P. concavitympanum* clade in South America. Inset map highliting the known geographic range of *Proceratophrys korekore* sp. nov. Abbreviations: MT, Mato Grosso state; PA, Pará state.

**Distribution.** The new species is known only from the type locality, Jacareacanga municipality, Pará state, and few sites on the other side of the Teles Pires River, Paranaíta municipality, Mato Grosso state (Fig. 8).

**Natural History.***Proceratophrys korekore* sp. nov. was found inhabiting only dense ombrophilous forest formations on both banks of the Teles Pires River. During nocturnal surveys between 18:00 and 21:00 h in the rainy season (from October to June), we observed males of *P. korekore* sp. nov. calling from the leaflitter on the banks of temporary streams. Some males were also found calling on bare soil near the edges of waterbodies. Additionally, individuals of the new species were observed during surveys (both diurnal and nocturnal) moving through the forest or captured using pitfall traps, even far from water bodies. Sympatric species include *Ameerega munduruku*, *Ceratophrys cornuta*, and *Lithodytes lineatus*. The main anthropogenic threats to the type locality are illegal logging and the often-subsequent deforestation for cattle grazing. Furthermore, illegal gold mining inside the forests (locally called “garimpo”) was observed during fieldwork. The activity of these illegal miners consists of clearing all vegetation of a target area and then destroying entire sections of stream bank to extract gold using mercury, a heavy metal that pollutes water supplies and poisons fish stocks (Silva et al., 2018). Finally, the Teles Pires River has also been dammed by multiple hydroelectric power plants, which have caused considerable natural habitat losses and fragmentation.

**Etymology.** The specific epithet “*korekore*” is a noun in apposition that means frog in the language of the Mundurukus, an indigenous group that inhabits the southwestern parts of Pará state and the northern region of Mato Grosso state, Brazil. We suggest the following Portuguese vernacular names “sapo-korekore” or “sapo-de-chifre-dos- mundurukus”.

## Discussion

The genus *Proceratophrys* has been historically arranged into three morphological groups (Giaretta, Bernarde & Kokubum, 2000; Kwet & Faivovich, 2001; Prado & Pombal, 2008; Amaro, Pavan & Rodrigues, 2009) without phylogenetic support (Amaro, Pavan & Rodrigues, 2009; Mângia et al., 2018). Resolving the phylogenetic relationships within *Proceratophrys* is essential to understand the genus’ diversity and evolutionary history. In previous studies on *Proceratophrys* using molecular data, only three clades were consistently recovered as monophyletic: the *P. bigibbosa* species group distributed in southern South America (Kwet & Faivovich, 2001), the clade formed by *P. cristiceps*, *P. minuta*, and *P. redacta* distributed in northeastern Brazil, and the *P. concavitympanum* clade composed of *P. concavitympanum*, *P. korekore* (Amazonia), *P. ararype* (Caatinga), *P. moratoi*, *P. salvatori* and *Proceratophrys* aff. *ararype* (Cerrado) (Amaro, Pavan & Rodrigues, 2009; Pyron & Wiens, 2011; Teixeira et al., 2012; Dias et al., 2013; Mângia et al., 2018; Magalhães et al., 2020; Cruz & Napoli, 2010). However, the relationships among the remaining sequenced (13 species) and not sequenced (15 species) species of *Proceratophrys* lack phylogenetic resolution and there are no morphological features that could be used to group them, highlighting the need for more robust phylogenetic analysis using multi-locus approaches and coalescent-based methods.

The population from Tocantins state, which we called *P*. aff. *ararype* here, has an average 1.8% genetic divergence in 16S from *P. ararype*. Genetic distance between species in the 16S barcode mtDNA locus is usually low within *Proceratophrys*, with some morphologically distinct species being less than 1.5% divergent (*e.g*., *P. belzebul-P. appendiculata-P. izecksohni*; *P. itamari-P. mantiqueira*) (Dias et al., 2013, Document S2). Nevertheless, based on the distributions of *P*. aff. *ararype* and *P. ararype* and no shared haplotypes among populations, further investigation is needed to clarify the taxonomic status of the population from Tocantins.

The description here of *P. korekore* sp. nov. constitutes the third new species described from the area under the influence of the São Manoel and Teles Pires hydroelectric power plants, the others being the watersnake *Helicops apiaka* (Kawashita-Ribeiro, Ávila & Morais, 2013) and the poison-frog *Ameerega munduruku* (Neves et al., 2017). These three taxa are known only from this region, which is also located in the ‘arc of deforestation’ (Souza et al., 2019). Habitat loss in Amazonia due to river dams and deforestation are directly causing vertebrate extinctions (Benchimol & Peres, 2015; Silva et al., 2018). This scenario underscores the need for sampling surveys and integrative taxonomic approaches to reveal Neotropical anuran diversity, which will lead to a better understanding of species distributions and evolutionary history.

## Appendix i

### Additional specimens examined

Acronyms: Coleção Herpetológica da Universidade Federal do Rio Grande do Norte (UFRN), Coleção Herpetológica da Universidade Federal de Pernambuco (CHUFPE), Museu de Zoologia da Universidade Federal da Bahia (MZUFBA), Coleção Zoológica da Universidade Federal de Mato Grosso (UFMT), Coleção Célio F. B. Haddad, Universidade Estadual Paulista (CFBH), Museu de Zoologia Prof. Adão José Cardoso, Universidade Estadual de Campinas (ZUEC), Museu de Zoologia da Universidade Estadual de Feira de Santana (MZFS), Coleção Herpetológica da Universidade Federal de Minas Gerais (CHUFMG), Museu de Ciências Naturais, Pontifícia Universidade Católica de Minas Gerais (MCNAM), Museu Nacional do Rio de Janeiro, Universidade Federal do Rio de Janeiro (MNRJ), and Coleção de Herpetologia da Universidade Regional do Cariri (URCAH).

*Proceratophrys appendiculata*.—BRAZIL: RIO DE JANEIRO: Angra dos Reis: MNRJ 34016. Guapimirim: MNRJ 30983. Nova Friburgo: MNRJ 34017. Rio de Janeiro: MNRJ 31547.

*Proceratophrys avelinoi*.—BRAZIL: PARANÁ: Guarapuava: MNRJ 33193–94. Nova Laranjeiras: MNRJ 30024–27.

*Proceratophrys belzebul*.—BRAZIL: SÃO PAULO: Ubatuba: MNRJ 87144.

*Proceratophrys boiei*.—BRAZIL: ALAGOAS: Murici: MNRJ 9719–20, 9726–29, 9732. Passo de Camaragibe: MNRJ 9817, 9862, 9863, 9864. Quebrangulo: MNRJ 9972. RIO DE JANEIRO: Teresópolis: MNRJ 37328–32.

*Proceratophrys branti*.—BRAZIL: TOCANTINS: Palmas: Taquarussu: UFMS AMP 5536–5538, 8118–8120; Novo Acordo: 8106.

*Proceratophrys brauni*.—BRAZIL: SANTA CATARINA: Timbé do Sul: MNRJ 25003–04.

*Proceratophrys carranca*.—BRAZIL: MINAS GERAIS: Buritizeiro: MNRJ 86440–42.

*Proceratophrys cristiceps*.—BRAZIL: ALAGOAS: Olho D’água do Casado: UFAL 8168-70. Piranhas: UFBA 8-9, 43. Traipu: Serra da Mão: UFAL 8968, 9035-36, 9043, 9196, 9510, 9656. BAHIA: Caetité: UFMG 5851. Campo Formoso: AAGARDA 12264-64, 12333-35. Jacobina: AAGARDA 12293-95; Paulo Afonso: UFPB 12114, 12119, 12122-23, 12128. CEARÁ: Aiuaba: AAGARDA 5111, 5132; URCA-H 7366, 7385, 7393, 7396, 7408, 7416, 7418. Barbalha: URCA-H 4293, 4571. Baturité: UFC3722. Crateús: URCA-H 4744. Crato: AAGARDA 2735, 2737-40. General Sampaio: UFC 5351. Itapipoca: AAGARDA 9817, 10453-55. Ipu: UFPB 6117-19, 6121, 6123, 6125. Jaguaribe: AAGARDA 10176-79, 10286, 10398-402. Milagres: MNRJ 55349, 55778-822, 75156-68; URCA-H 106, 142-43. Mucuripe: MNRJ 1419-20, 1680, 16470-84, 16487-89, 16591-600. Pacajus: UFC 4562. Paracuru: URCA-H 5773-74. Pentecoste: UFC 5001, 5018-19, 5193. São Gonçalo do Amarante: URCA-H 5669, 5775, 5860. Santa Quitéria: UFPB 10651, 10753-58. Serra das Almas: UFC 32, 131, 213, 224, 3319, 3464, 3467-68, 3470. Serra de Ibiapaba: UFPB 6117-26. Ubajara, Parque Nacional de Ubajara: AAGARDA 10672, 10695, 10698-99, 10703, 10707-09, 10782, 10796, 10907, 10909, 10911-14, 10961, 10974, 10981, 10983. Várzea da Conceição: UFPB 9661, 9665, 9667. PARAÍBA: Araruna: UFPB 8427, 8438, 8447, 8451, 8453, 8456, 8465, 8467, 8469, 8487. Boa vista: UFPB 1573-81. Cabaceiras: UFPB 6691-94, 11271, 11274. São José dos Cordeiros: UFPB 5866. PERNAMBUCO: Arcoverde: UFPB 9678-82, 9684, 9686-88, 9692, 9701. Betânia: UFC 3331. Bezerros: UFPB 7098. Exu: URCA 1462-63; UFPB 7214-17. Nascente: UFPB 9670. Ouricuri: URCA 2988-89. Buíque, Parque Nacional do Catimbau: AAGARDA 7706-12, 7747, 7760-61, 7765, 7799, 7802, 7804-05, 7824, 7886, 7975, 8056, 8362, 8417, 8435, 8437-40, 8450, 8463. Serra Talhada: UFPB 9656, 9659, 9660. Trindade: UFPB 974, 9673-77. PIAUÍ: Floriano: UFPI 214-16, 222, 236. Piripiri: UFPB 10340, 10342-46. RIO GRANDE DO NORTE: Serra Negra do Norte, Estação Ecológica do Seridó: AAGARDA 5447, 5528, 5583, 5689, 6061, 6790. João Câmara: AAGARDA 8913-15, 9806-11; URCA 422, 427, 483-85, 487-88, 493, 498, 501. Macaíba, Escola Agrícola de Jundiaí: AAGARDA 1013-14, 1019-20, 1753-71, 1773, 1776, 1778, 1786-91, 1935, 2495-96, 2583, 3757, 5447, 5528, 5554, 5583, 5689, 6061, 6790, 8866-71, 8913-15, 9806-11. SERGIPE: Poço Redondo: UFPB 12120-21, 12125-27.

*Proceratophrys concavitympanum*.—BRAZIL: MATO GROSSO: Aripuanã: MZUFV 9552, 9554–95556, UFMT 11697–11699; Colniza: UFMT 6808; Juína: UFMT 6996, 7825. RONDÔNIA: Espigão D’Oeste: CFBH 5135, 5136; Ministro Andreazza: CFBH 19815, CFBH 19818.

*Proceratophrys cururu*.—BRAZIL: MINAS GERAIS: Santana do Riacho: MNRJ 17905.

*Proceratophrys gladius*.—BRAZIL: SÃO PAULO: São José do Barreiro: MNRJ: 82577–79.

*Proceratophrys goyana*.—BRAZIL: GOIÁS: Colinas do Sul: MNRJ 68292–95. Minaçu: MNRJ 17309–14. Rio São Miguel: MNRJ 47902. Veadeiros: MNRJ 47901, 47903– 04.

*Proceratophrys itamari*.—BRAZIL: SÃO PAULO: Campos do Jordão: MNRJ 82580–84.

*Proceratophrys izecksohni*.—BRAZIL: RIO DE JANEIRO: Parati: MNRJ 88985–86.

*Proceratophrys korekore* sp. nov.—BRAZIL: MATO GROSSO: Paranaíta: CFBH 20675–20678, ZUFMS-AMP8100–8101, 8103, 8105, 8116, UFMT 7534, 7963, 9882, 9990, 10038, 10041, 10046, 10054, 10067, 10109, ZUEC 14874–14876, 16011–16015, 16719. PARÁ: Jacareacanga: ZUEC 14950; MNRJ 90237

*Proceratophrys laticeps*.—BRAZIL: BAHIA: Ilhéus: MNRJ 4124–26, 13950–55. ESPÍRITO SANTO: Conceição da Barra: MNRJ 27946, 27949.

*Proceratophrys mantiqueira*.—BRAZIL: MINAS GERAIS: Ervália: MNRJ 82573–76.

*Proceratophrys melanopogon*.—BRAZIL: RIO DE JANEIRO: Resende: MNRJ 51654–705.

*Proceratophrys minuta*.—BRAZIL: BAHIA: Miguel Calmon, Parque Estadual das Sete Passagens: MNRJ 75410–17.

*Proceratophrys moehringi*.—BRAZIL: ESPÍRITO SANTO: Santa Teresa: MNRJ 46804.

*Proceratophrys moratoi*.—BRAZIL: SÃO PAULO: Botucatu: MNRJ 60085.

*Proceratophrys paviotii*.—BRAZIL: ESPÍRITO SANTO: Santa Teresa: MNRJ 84079–80; Aracruz: MNRJ 40182–84.

*Proceratophrys renalis*.—BRAZIL: ALAGOAS: Passo de Camaragibe: MNRJ 9817.

*Proceratophrys rondonae*.—BRAZIL: RÔNDONIA: Bacia do Rio Branco: MNRJ 40906.

*Proceratophrys sanctaritae*.—BRAZIL: BAHIA: Amargosa: MNRJ 62354–62357.

*Proceratophrys schirchi*.—BRAZIL: BAHIA: Guaratinga: MNRJ 26459–60; Jussari: MNRJ 26456–58; Nilo Peçanha: MNRJ 26461. ESPÍRITO SANTO: Santa Teresa: MNRJ 18445–46, 56000–01.

*Proceratophrys strussmannae*.—BRAZIL: MATO GROSSO: Vale de São Domingos: UFMT 1834, 1836, 7882, 7885, 8319,8320, 8377, 8380; Araputanga: UFMT 7879.

*Proceratophrys subguttata*.—BRAZIL: SANTA CATARINA: Brusque: MNRJ 18282; São Bento do Sul: MNRJ 18281.

*Proceratophrys tupinamba*.—BRAZIL: RIO DE JANEIRO: Angra dos Reis: MNRJ 25101–18, 38938.

*Proceratophrys vielliardi*.—BRAZIL: GOIÁS: Caldas Novas: MNRJ 83314–15.

## Supplemental Information

10.7717/peerj.12012/supp-1 Supplemental Information 1 Genbank acession number and references of the sequences used in the present work.

10.7717/peerj.12012/supp-2 Supplemental Information 2 Uncorrected p-distances for a 497-bp aligned sequence of the 16S gene of the new species and other Proceratophrys species taken from GenBank (see SM 1). Data in bold are mean intraspecific divergences between P. korekore sp. nov. and the other species.

10.7717/peerj.12012/supp-3 Supplemental Information 3 16s sequence alignment.

## Additional Information and Declarations

### Field Study Permissions

The following information was supplied relating to field study approvals (*i.e*., approving body and any reference numbers): Collection permits for this study were issued by ICMBIO (SISBio 45889-1).

### DNA Deposition

The following information was supplied regarding the deposition of DNA sequences: The 16S mtDNA sequences described here are available at GenBank: MW889928 to MW889930.

### Data Availability

The following information was supplied regarding data availability: Data are available at GitHub: https://github.com/Rhinella85/Proceratophrys_korekore.git All specimens used in the article are stored at: Coleção Zoológica da Universidade Federal de Mato Grosso do Sul (acronym ZUFMS-AMP): ZUFMS-AMP08100 (Holotype), ZUFMS-AMP08098, ZUFMS-AMP08099, ZUFMS-AMP08101, ZUFMS-AMP08103, ZUFMS-AMP08102, ZUFMS-AMP08104, ZUFMS-AMP08105, ZUFMS-AMP08116, ZUFMS-AMP08117, ZUFMS-AMP13680, ZUFMS-AMP13681 Coleção de Anfíbios Célio Fernando Batista Haddad (acronym CFBH): CFBH20675, CFBH20676, CFBH20678, CFBH20677 Coleção Zoológica da Universidade Federal de Mato Grosso (acronym UFMT-A): UFMT-A7963, UFMT-A9882, UFMT-A10038, UFMT-A9990, UFMT-A10041, UFMT-A10046, UFMT-A10054, UFMT-A10067, UFMT-A10109 Museu de Zoologia da Universidade Estadual de Campinas “Adão José Cardoso” (ZUEC): ZUEC14874, ZUEC14875, ZUEC14950, ZUEC14876, ZUEC16011, ZUEC16012, ZUEC16013, ZUEC16014, ZUEC16719, UFMT-A7534

### New Species Registration

The following information was supplied regarding the registration of a newly described species: Publication LSID: urn:lsid:zoobank.org:pub:4077F2CC-A0B1-49AC-B562-2FE7CC929668 *Proceratophrys korekore* LSID: urn:lsid:zoobank.org:act:53DF6075-CA41-4FAD-88BB-E26A9B6BF67F.
